# Supplementary material for: Markers of prolonged hospitalisation in severe dengue
Source: PLoS Negl Trop Dis. 2024 Jan 30;18(1):e0011922. doi: 10.1371/journal.pntd.0011922 (PMC10857710; doi:10.1371/journal.pntd.0011922)
Supplement: S1 Table — (PDF) [file pntd.0011922.s001.pdf]

Markers of prolonged hospitalisation in severe dengue: a retrospective cohort study

Supplementary Material

Table 1: Overview of patient characteristics and disease markers at time of hospitalisation.

	1 (N=1238)	2 (N=355)	3 (N=21)	Total (N=1614)	p value
**Year**					0.042
2017	935 (75.5%)	258 (72.7%)	14 (66.7%)	1207 (74.8%)	
2018	162 (13.1%)	38 (10.7%)	2 (9.5%)	202 (12.5%)	
2019	141 (11.4%)	59 (16.6%)	5 (23.8%)	205 (12.7%)	
Missing	0	0	0	0	
**Age**					0.002
Mean (CI)	40 (39, 41)	36 (34, 37)	37 (30, 44)	39 (38, 39)	
Range (Min - Max)	13 - 91	13 - 78	17 - 68	13 - 91	
Missing	0	0	0	0	
**Sex**					0.441
female	605 (48.9%)	187 (52.7%)	10 (47.6%)	802 (49.7%)	
male	633 (51.1%)	168 (47.3%)	11 (52.4%)	812 (50.3%)	
Missing	0	0	0	0	
**Change.severity**					< 0.001

	1 (N=1238)	2 (N=355)	3 (N=21)	Total (N=1614)	p value
0	1238 (100.0%)	355 (100.0%)	21 (100.0%)	1614 (100.0%)	
Missing	0	0	0	0	
**Day of illness**					< 0.001
Mean (CI)	4 (3, 4)	5 (5, 5)	5 (4, 6)	4 (4, 4)	
Range (Min - Max)	1 - 10	1 - 8	1 - 10	1 - 10	
Missing	0	0	0	0	
**Hospital stay [days]**					< 0.001
Mean (CI)	6 (5, 6)	5 (5, 5)	8 (6, 10)	5 (5, 6)	
Range (Min - Max)	1 - 18	1 - 15	1 - 18	1 - 18	
Missing	0	0	0	0	
**Dengue.Infection.History**					0.604
no	1224 (99.4%)	351 (98.9%)	21 (100.0%)	1596 (99.3%)	
yes	8 (0.6%)	4 (1.1%)	0 (0.0%)	12 (0.7%)	
Missing	6	0	0	6	
**Pulse**					< 0.001
Mean (CI)	90 (89, 91)	86 (85, 87)	91 (82, 99)	89 (89, 90)	
Range (Min - Max)	55 - 150	56 - 120	60 - 140	55 - 150	
Missing	15	1	1	17	
**Temperature**					< 0.001
Mean (CI)	38 (38, 38)	38 (38, 38)	38 (38, 39)	38 (38, 38)	
Range (Min - Max)	35 - 41	35 - 41	36 - 40	35 - 41	
Missing	31	5	1	37	
**Systolic blood pressure**					< 0.001
Mean (CI)	114 (114, 115)	111 (110, 112)	104 (98, 111)	114 (113, 114)	
Range (Min - Max)	80 - 181	80 - 170	65 - 125	65 - 181	

	1 (N=1238)	2 (N=355)	3 (N=21)	Total (N=1614)	p value
Missing	15	0	1	16	
**Diastolic blood pressure**					0.084
Mean (CI)	71 (70, 71)	70 (69, 71)	66 (62, 71)	71 (70, 71)	
Range (Min - Max)	50 - 130	40 - 100	40 - 80	40 - 130	
Missing	15	0	1	16	
**Headache**					0.787
no	27 (2.2%)	8 (2.3%)	0 (0.0%)	35 (2.2%)	
yes	1197 (97.8%)	347 (97.7%)	21 (100.0%)	1565 (97.8%)	
Missing	14	0	0	14	
**Body ache**					0.673
no	37 (3.0%)	12 (3.4%)	0 (0.0%)	49 (3.1%)	
yes	1187 (97.0%)	343 (96.6%)	21 (100.0%)	1551 (96.9%)	
Missing	14	0	0	14	
**Fatigue**					0.057
no	346 (28.0%)	121 (34.1%)	8 (38.1%)	475 (29.4%)	
yes	891 (72.0%)	234 (65.9%)	13 (61.9%)	1138 (70.6%)	
Missing	1	0	0	1	
**Bleeding_any**					< 0.001
no	977 (78.9%)	87 (24.5%)	7 (33.3%)	1071 (66.4%)	
yes	261 (21.1%)	268 (75.5%)	14 (66.7%)	543 (33.6%)	
Missing	0	0	0	0	
**Rash**					0.004
no	1176 (96.2%)	327 (92.1%)	19 (90.5%)	1522 (95.2%)	
yes	47 (3.8%)	28 (7.9%)	2 (9.5%)	77 (4.8%)	
Missing	15	0	0	15	
**Ascites**					< 0.001
no	1221 (99.9%)	355 (100.0%)	19 (95.0%)	1595 (99.9%)	
yes	1 (0.1%)	0 (0.0%)	1 (5.0%)	2 (0.1%)	

	1 (N=1238)	2 (N=355)	3 (N=21)	Total (N=1614)	p value
Missing	16	0	1	17	
**Vomiting**					< 0.001
no	1083 (88.5%)	284 (80.0%)	9 (45.0%)	1376 (86.1%)	
yes	141 (11.5%)	71 (20.0%)	11 (55.0%)	223 (13.9%)	
Missing	14	0	1	15	
**Liver enlargement**					< 0.001
no	1216 (99.3%)	340 (95.8%)	19 (95.0%)	1575 (98.5%)	
yes	8 (0.7%)	15 (4.2%)	1 (5.0%)	24 (1.5%)	
Missing	14	0	1	15	
**Oliguria**					0.858
no	1223 (99.9%)	355 (100.0%)	20 (100.0%)	1598 (99.9%)	
yes	1 (0.1%)	0 (0.0%)	0 (0.0%)	1 (0.1%)	
Missing	14	0	1	15	
**Diabetes**					0.133
no	1190 (96.6%)	350 (98.6%)	20 (95.2%)	1560 (97.0%)	
yes	42 (3.4%)	5 (1.4%)	1 (4.8%)	48 (3.0%)	
Missing	6	0	0	6	
**Hepatitis.B**					0.168
no	1218 (98.8%)	347 (97.7%)	20 (95.2%)	1585 (98.5%)	
yes	15 (1.2%)	8 (2.3%)	1 (4.8%)	24 (1.5%)	
Missing	5	0	0	5	
**Gastritis**					0.431
no	1171 (95.0%)	343 (96.6%)	20 (95.2%)	1534 (95.3%)	
yes	62 (5.0%)	12 (3.4%)	1 (4.8%)	75 (4.7%)	
Missing	5	0	0	5	
**Pregnancy**					< 0.001
no	681 (90.3%)	232 (99.1%)	14 (93.3%)	927 (92.4%)	

	1 (N=1238)	2 (N=355)	3 (N=21)	Total (N=1614)	p value
yes	73 (9.7%)	2 (0.9%)	1 (6.7%)	76 (7.6%)	
Missing	484	121	6	611	
**NS1**					0.162
Mean (CI)	1 (1, 1)	1 (1, 1)	1 (-1, 2)	1 (1, 1)	
Range (Min - Max)	0 - 1	0 - 1	0 - 1	0 - 1	
Missing	917	291	18	1226	
**IgG**					
Mean (CI)	1 (-0, 2)	NA	1 (NaN, NaN)	1 (0, 1)	
Range (Min - Max)	0 - 1	NA	1 - 1	0 - 1	
Missing	1234	355	20	1609	
**IgM**					0.029
Mean (CI)	0 (0, 0)	0 (0, 0)	1 (-1, 2)	0 (0, 0)	
Range (Min - Max)	0 - 1	0 - 1	0 - 1	0 - 1	
Missing	919	294	18	1231	
**Leucocytes (WBC)**					< 0.001
Mean (CI)	6 (5, 6)	4 (3, 4)	6 (3, 10)	5 (5, 6)	
Range (Min - Max)	0 - 82	1 - 8	3 - 10	0 - 82	
Missing	887	277	16	1180	
**Neutrophils (NEU)**					< 0.001
Mean (CI)	67 (65, 69)	50 (46, 54)	55 (25, 85)	64 (62, 66)	
Range (Min - Max)	15 - 92	14 - 87	38 - 72	14 - 92	
Missing	887	278	17	1182	
**Lymphocytes (LYM**					< 0.001
Mean (CI)	19 (18, 21)	31 (27, 34)	29 (11, 47)	22 (20, 23)	
Range (Min - Max)	2 - 68	5 - 67	15 - 52	2 - 68	
Missing	887	278	16	1181	

	1 (N=1238)	2 (N=355)	3 (N=21)	Total (N=1614)	p value
**Erythrocytes (RBC)**					< 0.001
Mean (CI)	5 (4, 5)	5 (5, 5)	4 (3, 5)	5 (5, 5)	
Range (Min - Max)	3 - 7	4 - 6	3 - 4	3 - 7	
Missing	887	278	16	1181	
**Hemoglobin (Hb)**					0.003
Mean (CI)	134 (132, 136)	139 (136, 143)	114 (90, 139)	135 (133, 136)	
Range (Min - Max)	76 - 178	92 - 171	81 - 130	76 - 178	
Missing	887	278	16	1181	
**Hematocrit (HCT)**					0.004
Mean (CI)	0 (0, 0)	0 (0, 0)	0 (0, 0)	0 (0, 0)	
Range (Min - Max)	0 - 1	0 - 1	0 - 0	0 - 1	
Missing	887	278	16	1181	
**Platelets (PLT)**					< 0.001
Mean (CI)	142 (136, 149)	73 (61, 85)	84 (6, 161)	129 (123, 136)	
Range (Min - Max)	11 - 371	7 - 210	31 - 190	7 - 371	
Missing	887	278	16	1181	
**Prothrombin (PT_s)**					0.058
Mean (CI)	16 (15, 16)	14 (14, 15)	14 (12, 16)	15 (15, 16)	
Range (Min - Max)	13 - 22	11 - 18	13 - 15	11 - 22	
Missing	1192	337	18	1547	
**Prothrombin (PT_pc)**					0.223
Mean (CI)	82 (77, 87)	93 (81, 104)	82 (64, 100)	85 (81, 90)	
Range (Min - Max)	51 - 120	68 - 162	76 - 90	51 - 162	
Missing	1192	337	18	1547	
**International Normalized Ratio (INR)**					0.259
Mean (CI)	1 (1, 1)	1 (1, 1)	1 (1, 1)	1 (1, 1)	
Range (Min - Max)	1 - 2	1 - 1	1 - 1	1 - 2	
Missing	1193	338	18	1549	

	1 (N=1238)	2 (N=355)	3 (N=21)	Total (N=1614)	p value
**Fibrinogen (Fib)**					0.232
Mean (CI)	3 (3, 3)	2 (-0, 4)	2 (NaN, NaN)	3 (2, 3)	
Range (Min - Max)	1 - 5	1 - 3	2 - 2	1 - 5	
Missing	1210	352	20	1582	
**Activated Partial Thromboplastin Time (APTT)**					0.003
Mean (CI)	35 (32, 38)	42 (39, 46)	47 (-48, 142)	37 (35, 40)	
Range (Min - Max)	1 - 54	35 - 56	39 - 54	1 - 56	
Missing	1204	342	19	1565	
**Glucose (Glu)**					0.920
Mean (CI)	7 (6, 7)	8 (5, 11)	7 (-10, 24)	7 (6, 7)	
Range (Min - Max)	-3 - 17	4 - 72	0 - 14	-3 - 72	
Missing	1012	306	18	1336	
**Uric acid (Ure)**					0.854
Mean (CI)	4 (4, 4)	4 (4, 5)	5 (1, 9)	4 (4, 4)	
Range (Min - Max)	1 - 8	2 - 8	3 - 8	1 - 8	
Missing	1021	312	17	1350	
**Creatinin (Cre)**					0.254
Mean (CI)	120 (51, 188)	80 (75, 85)	82 (75, 89)	112 (57, 168)	
Range (Min - Max)	4 - 8101	49 - 136	77 - 86	4 - 8101	
Missing	1007	306	17	1330	
**Albumin (Alb)**					0.033
Mean (CI)	40 (38, 42)	38 (31, 45)	25 (NaN, NaN)	40 (38, 42)	
Range (Min - Max)	27 - 93	1 - 95	25 - 25	1 - 95	
Missing	1163	334	20	1517	
**Aspartate Aminotransferase (AST)**					< 0.001
Mean (CI)	67 (57, 77)	162 (110, 213)	1030 (453, 1607)	97 (79, 116)	
Range (Min - Max)	13 - 576	11 - 819	530 - 1324	11 - 1324	
Missing	1001	301	17	1319	

	1 (N=1238)	2 (N=355)	3 (N=21)	Total (N=1614)	p value
**Alanine Aminotransferase (ALT)**					< 0.001
Mean (CI)	50 (40, 60)	103 (70, 137)	542 (106, 978)	66 (54, 78)	
Range (Min - Max)	5 - 937	8 - 550	199 - 869	5 - 937	
Missing	994	301	17	1312	
**Gamma-Glutamyl Transferase (GGT)**					0.002
Mean (CI)	53 (39, 66)	108 (56, 159)	123 (-57, 303)	63 (49, 77)	
Range (Min - Max)	7 - 888	5 - 778	60 - 202	5 - 888	
Missing	1051	315	18	1384	
