# Supplementary figures and images for: Markers of prolonged hospitalisation in severe dengue
Source: PLoS Negl Trop Dis. 2024 Jan 30;18(1):e0011922. doi: 10.1371/journal.pntd.0011922 (PMC10857710; doi:10.1371/journal.pntd.0011922)

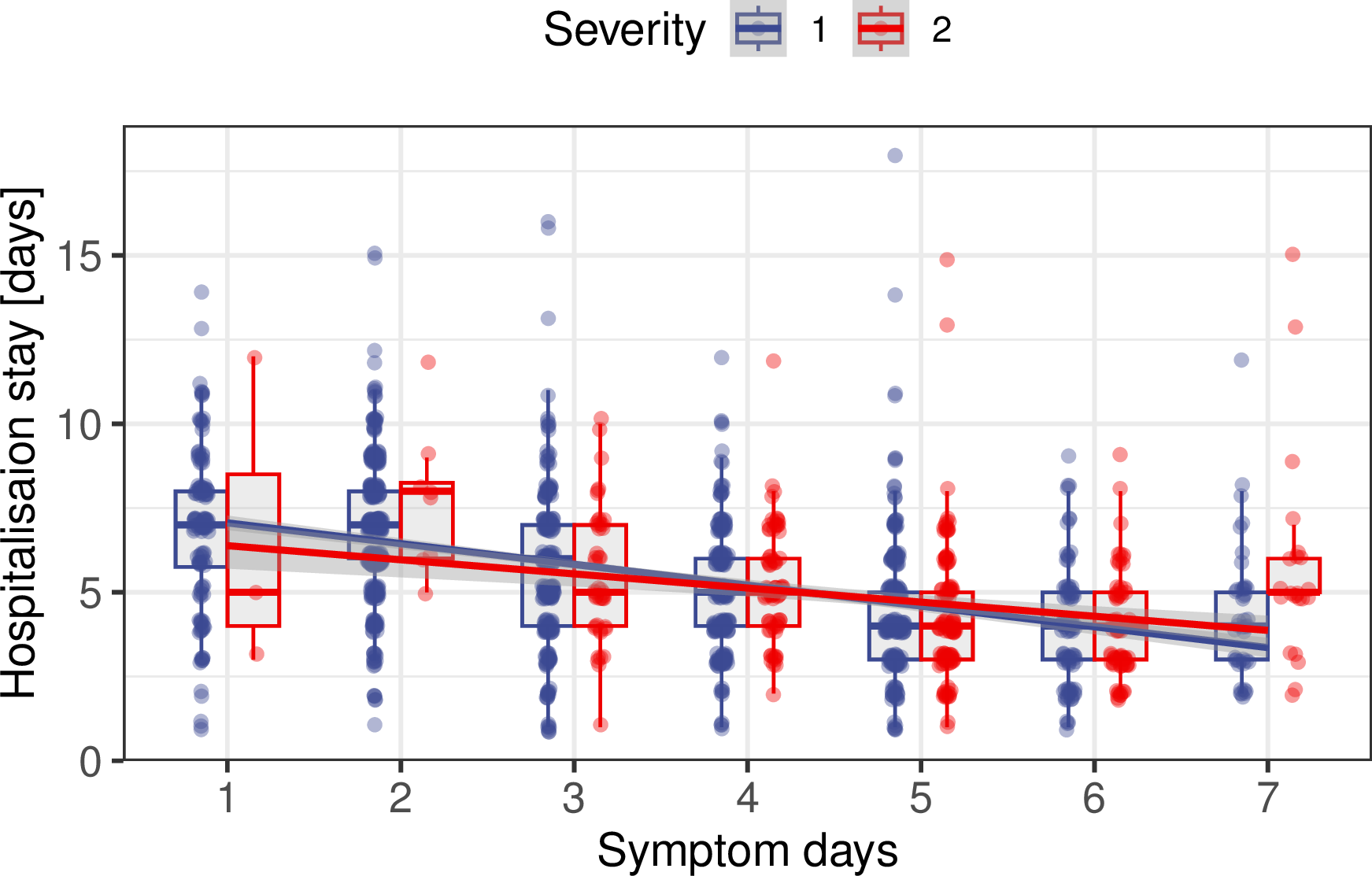

Supplement: S1 Fig — Duration of hospitalisation is negatively correlated with the reported number of days since symptom onset prior to hospitalisation, irrespective of diagnosed disease severity. (TIF) [file pntd.0011922.s002.tif]

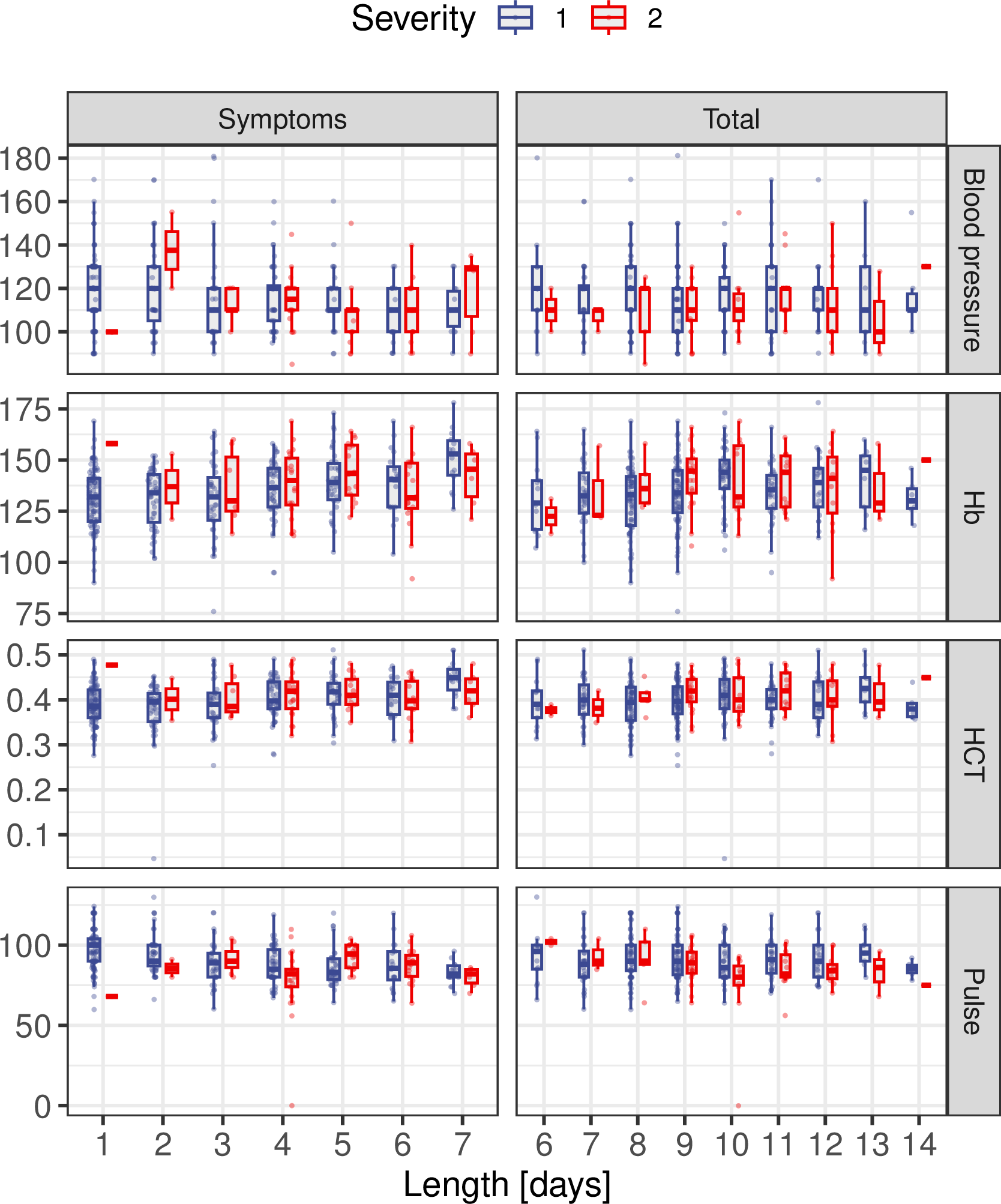

Supplement: S2 Fig — Four diagnostic markers (systolic blood pressure, pulse, haemoglobin (Hb) and haematocrit (HCT)) show different correlations with pre-hospitalisation symptom days with little difference between disease severity scores 1 and 2. Any temporal correlations disappear when regressing against the total number of disease days, leading to more pronounced differences between dengue severity scores. (TIF) [file pntd.0011922.s003.tif]
