# Supplementary material for: Markers of prolonged hospitalisation in severe dengue
Source: PLoS Negl Trop Dis. 2024 Jan 30;18(1):e0011922. doi: 10.1371/journal.pntd.0011922 (PMC10857710; doi:10.1371/journal.pntd.0011922)
Supplement: Attachment — Submitted filename: response_to_reviewers.doc [file pntd.0011922.s005.doc]

**Response to reviewers comment**

We would like to thank the reviewers for their thoughtful comments, which we believe has helped to make this a more robust analysis of dengue hospitalisation data. We would also like to apologise for the length of time it has taken to prepare the revised manuscript. Following the queries raised by the reviewers regarding the dengue severity score, how this was defined and whether this changed during a patients stay in hospital, we went through all patient records again to access the severity score assigned at admission, rather than the *highest* severity score recorded, as was the case in the original analysis. We now only consider patients with a recorded severity score assigned at admission and exclude all patients whose severity score changed during their hospital stay. We reran all of our analyses, and although the total number of patients analysed is now smaller, it is still sufficiently large to make robust inferences, which we are happy to report have not changed.

A point-by-point response to all reviewers’ comments is provided below.

**Reviewer #1:**

Methods:

1. To analyse the risk factors associated with duration of hospitalization, did the authors consider to evaluate presence of comorbidies: diabetes, obesity etc… Was disease severity at time of admission to hospital recorded and analysed? i.e. how many had dengue with warning signs or DHF when they were admitted?

Unfortunately we do not have sufficient information on patient co-morbidities in our dataset, although we agree that this would be an interesting, and potentially important, covariate for our analysis. With regards to disease severity, and as mentioned above, this was recorded at the time of hospital admission. Of the 1614 patients we considered in this analysis (excluding those who died), 1238 had a severity score of 1, 355 had a severity score of 2, and only 21 patients had a severity score of 3. Although on severity scores 1 and 2 are considered in the manuscript; Supplementary Table 1 provides an overview of all patients.

2. As the readers are not familiar with disease severity score of 1 to 3, can a brief summary be given? Otherwise its very difficult to interpret and understand the data

We fully agree and now provide a detailed summary of the clinical criteria for assigning different severity scores.

3. The number of patients classifies has disease severity score of 1 and 2 is less than the total number of patients. The numbers don’t add up.

Thank you for pointing this out. Unfortunately there was an error reporting the total number of patients. We have clarified this in the revised manuscript and made sure that they all add up now.

Results

1. the overview of patient characteristics does not provide much data. What about the laboratory parameters? Also could the authors list the symptoms and the proportion of symptoms at the time of presentation

We have now extended Table 1 to include further diagnostic markers and symptoms and a comprehensive overview about all lab and symptom data at the time of presentation to hospital is provided as Supplementary Information (referenced in the main text).

2. 63% of patients having bleeding manifestations is alarming. Rather than the %, can the actual numbers be given. The numbers don’t add up in many parts of the manuscript.

The reason why we did not report numbers here, in addition or instead of percentages, is because we did not have full information for all patients. As the reviewer pointed out, some of the numbers appear not to add up – due to missingness in our dataset – which would be exacerbated by providing different denominators for different symptoms. Data table 1 now also provides information about the degree of missingness in the datas.

3. The patients with severe disease had longer hospitalization? Is this because those who presented late to hospital, had a delay in management (i.g. fluid therapy) and therefore, already had severe disease at the time of hospitalization. It is important to provide clinical disease severity at presentation, as it is not possible to make sense of the data.

As mentioned earlier, in this analysis we only consider disease severity as recorded at the time of hospital admission and discarded those whose severity score changed over their hospital stay. Regarding the length of hospitalisation, here we report a negative relationship, such that patients with a higher severity score (2 vs. 1) had, on average, shorter hospital stays.

General comments:

there seem to be quite a bit of missing data due to the retrospective nature of the study. It would be important to analyse the risk factors for prolonged hospitalization and this has not been addressed in this study adequately. As a result of this, many of the interpretations of results, don’t seem to be rational.

Unfortunately we are unsure what analyses the reviewer is referring to here. It is true that data was missing (not all risk factors were recorded for all patients); however, given the high number of patients we believe that our inferences about the various risk factors are robust. The aim of this study was to analyse risk factors for prolonged hospitalisation, and here we show how the time to admission (from symptom onset) is one of the strongest predictors. We would therefore like to ask for further clarification which of our interpretations do not seem to be rational.

Reviewer #2

The authors report their analysis of a dataset collected over several years in Vietnam looking at risk factors/prognostic markers that may determine length of hospital stay and potentially be useful for healthcare planning/resource allocation. Their main finding is of an “unexpected correlation between markers of disease severity and hospitalisation length, which can be resolved by taking the period of dengue symptoms prior to hospitalisation into account”. However this is not actually unexpected!

The first major point is that the natural evolution of dengue signs, symptoms and laboratory results is well known and many observational/descriptive studies and clinical trials already take the period of time from illness onset into account – with analyses described by fever day, illness day etc. The quite extensive existing literature should be acknowledged and summarised. Specifically with respect to viremia and primary/secondary infections (as mentioned in the discussion) there are several publications, including a very large dataset (Vuong NL et al. CID, 2021) that look at these data by day of illness and show relationships with markers of clinical severity.

The value of the work presented is in corroborating this well-known fact and showing mathematically that taking the parameter referred to here as “symptom day” into account has a major influence on the various biomarkers assessed.

The reviewer is correct in that several studies have previously shown the evolution of disease and disease markers since onset of symptoms, and we agree that this was perhaps not adequately covered in our manuscript (we now refer to this in the Discussion of our revised manuscript). As far as we are aware, though, the relationship between disease markers, dengue severity and subsequent length of hospitalisation has only scarcely been covered, and we honestly believe that this is one of the first that reports on this association. It is entirely possible that we have missed some key literature, however, and we would be grateful if the reviewer could provide further references.

The second major point is that the authors cannot really comment on relationships with severity when they exclude all death cases and those with severity score 3. The remaining patients are effectively those with and without warning signs, and since there is a lot of missing data even within these groups it is not surprising that relationships to severity were difficult to identify. Please include a section on the quality of the data (degree of missingness) in the main text and comment on how this might have influenced the results. Also comment on the exclusion of all severe cases…..

We kindly disagree with this statement. Of the 1614 patient that we considered here (excluding 5 patients who died and which we only had minimal information for), only 21 had a severity score of 3, so we are unsure how these could influence any of the relationships that we report. However, we believe that some confusion might be due to the wording of ‘severity’, which in this report we refer to in the context of the assigned ‘dengue severity score’, which, as the reviewer correctly points out, distinguishes between dengue without and with warning signs (severity score 1 and 2, respectively) and ‘severe’ dengue (severity score 3). We have clarified this in the revised version where we also provide more details on the criteria used to assign severity scores.

In addition, the now extended Table 1 and Supplementary Table 1 provide the degree of missingness for each of the variables under consideration (note: different markers had different degrees of missingness). We now also now state our rationale for excluding those patients with a recorded severity score of 3, of those who died and those with extended hospitalisation periods (>21 days).

Many factors other than clinical severity determine when an individual is hospitalised (some of which are mentioned in the text), but also when they are discharged. Discharge guidelines (often including arbitrary lab values rather than focusing on clinical severity markers), the need for beds for other patients, the day of the week (staff are often unavailable at weekends to complete discharge papers so discharges are more common on Fridays and Mondays) etc. etc. A more detailed discussion of the factors that may affect both admission and discharge days is warranted, as well as mention of the limited utility of length of hospitalisation as an indicator of clinical severity.

We fully agree with this statement, but unfortunately, information on hospital discharge and determinants thereof was not available to us so we really cannot comment on those. We agree that many different factors will influence the decision of discharging a patient or not and that these may well vary by time and between healthcare settings, as already alluded to in the Discussion and also shown in Figure 5B. However, without any more information these remain speculative, and unless there is a reason to suspect that they have introduced a systematic bias in our analysis, we do not think that further discussion is warranted. Please also note that the length of hospitalisation was not considered an indicator of clinical severity in our analysis.

It is not clear to me whether all the clinical/lab data analysed was from the first assessment only? Please clarify exactly which data are included. Also for the outcome severity scoring please include more specific details of how this was done, potentially in the appendix. How many assessments were required to be able to give a score to an individual? What happened if key variables were missing?

We apologise that this information as not entirely clear. We only consider clinical / lab data from the initial assessment, as mentioned in the Methods section, and we now provide further detail on the criteria used to assign a particular severity score in the Methods section of our revised manuscript.

The author summary refers to a “delay in admission in those patients with higher severity scores”. This interpretation of the data, and specifically use of the word “delay” is unwarranted and potentially problematic for health services in endemic settings. There are many reasons why slightly more symptomatic cases may be overrepresented among the later admissions, but there is nothing to suggest that the outcome would have been any different if they had been admitted earlier. We know that a huge proportion of mildly symptomatic dengue cases never present to clinical services at all – maybe the more stoical among them were feeling better by day 3/4/5, meaning that the majority of those who presented to a health facility at this time were those who felt a bit worse or were more worried than their counterparts. Suggesting that all these individuals should have been admitted earlier could increase the burden on health services

We fully agree with this statement; however, at no point in our manuscript do we suggest that earlier hospitalisation would affect the outcome as such or argue that individuals should be admitted earlier. If anything, our analysis suggest that later hospitalisation is, on average, associated with shorter durations. However, we agree that the word ‘delay’ could easily be misinterpreted and have therefore removed references to it in our revised manuscript except in cases where a misunderstanding is highly unlikely.

**Reviewer #3**

General:

This is a well written manuscript on an important topic. The data set used seems to be promising and the methodology is sound. However, there seems to be a lack of reference to the natural history of the disease and its features over time – for example the onset of the ‘critical period’ at around day of illness 4-6 (with more severe disease for a subset of Dengue patients).

It seems that the authors conclude – as one of their findings - that it is important to take into account ‘total illness days’ (the authors also call this a ‘lead time bias’). It is well known in the field that ‘day of illness’ is an important variable for the clinical evaluation of dengue patients and that depending on this, the presence or absence of certain clinical signs and symptoms (e.g., ‘warning signs’) has to be interpreted differently. Thus, adjusting for ‘day of illness’ is not a new finding and results that don’t take into account ‘day of illness’ might be misunderstood. This leads to a situation where the findings of this manuscript don’t integrate well with the body of literature. This reviewer believes that if the analysis would take into account (adjust for/stratify by) ‘day of illness’ from the very start, the findings would be much easier to interpret.

This is a very important point. Although it might well be the case that day of illness is an important variable for clinical evaluation, we have not come across a study where potential biomarkers for hospitalisation length have been stratified by day of illness and disease severity scores. The aim of this study was therefore to show how different markers of disease and their associations with shorter or longer stays is influenced by illness days, rather than assigned disease severity. We believe that this is an important message to reiterate and therefore feel that the current structure is preferred over one starting off with a stratified analysis.

In many countries in Southeast Asia (presumably also in Vietnam), patients try to stay at home as long as they can manage and are only hospitalized for dengue if more severe disease or complications are suspected, either as a result of dengue itself (which usually happens around day 4-6, in the ‘critical period’) or as a result of existing comorbidities or difficulties at home (being alone, living too far from a health facility). This means that around day of illness 3-5, a proportion of the patients with symptomatic Dengue actually get better and are never hospitalized. The ‘pool’ of patients that proceeds to more severe disease is smaller starting at around day of illness 3-5, but it includes the patients that have a higher probability of more severe disease.

This is another important point and is also exactly what we find in the data (Figure 1B), individuals with severity score 2 get on average hospitalised at a later stage than individuals with severity score 1. And we also agree that the pool / demographic of individuals could likely be different between those with severity 1 or 2; unfortunately, however, we do not have any further information to allow us to delve deeper into this but have added this as a potential explanation to the Discussion of our revised manuscript. One thing to note, our analysis shows that in terms of measured biomarkers, some of the difference between those with severity score 1 and 2 can be explained by the difference in the time to hospital admission.

I hope that these introductory remarks help and below are more specific comments for the authors. I would recommend major revisions.

Thank you, these comments are very helpful and appreciated.

Background:

-	Line 100: “Our analysis reveals…” – should this not go into the results?

It is not uncommon to include a brief summary of the main findings at the end of the Introduction, so we believe this should be OK.

Methods:

-	Can you please include how dengue was confirmed by laboratory diagnosis?

We apologise that this information was missing, details on initial diagnosis and assignment of a severity score have now been added to the Methods section.

-	Line 109: Why was only the first blood draw analysed per patient? Were repeated blood results available per patient? What is the day of illness distribution at time of hospitalization / enrolment? Can you please include this information into table 1? Later in the manuscript (caption figure 4) it becomes clear that diagnostic markers show a strong correlation with day of illness…

The reason why we only analysed the first blood draw is because we wanted to understand how markers at first presentation could provide information about subsequent hospital stay. Although more data (from repeated blood samples) is available, their analysis is beyond the scope of the current manuscript.

Regarding the distribution of symptom days (before hospital admission), this is shown in Figure 1B, stratified by sex and severity score.

-	Line 113: What was the justification to restrict the analysis to the variables described at this point? Was there an a-priori analysis plan? Who decided on this set of variables?

We agree that information on this was crucially missing. These variables were selected either based on known or identified association with disease severity and also where we had sufficient data entries to derive meaningful inferences. We then used those variables to highlight how the observed associations are affected when taking symptom days into consideration. We have now expanded the Supplementary Material to include similar analyses of all variables that showed a significant association with severity.

-	Line 122/123: Can you provide more information about the adaptation of the WHO severity score by the Vietnamese Ministry of Health? This could go into the appendix.

We now provide full details on WHO criteria for dengue severity in the revised Methods section.

Table 1:

-	75% of the data comes from 2017. Can you stratify outcomes by year in table 1? Was a heterogeneity assessment conducted?

Although we agree that stratification by year is important to highlight inter-annual variations (as also shown in Fig. 5), Table 1 is already stratified by severity score, such that additional stratification by year would make this table quite ‘unwieldy’.

Results:

-	Line 151: The authors report about a potential non-linear relationship of age with the outcome. At a later point, age was modelled linear. Were you considering introducing splines for age?

The nonlinear relationship that we refer to is with regards to the age distribution of hospitalised individuals when stratified by sex and severity (which shows bi-modality for females) but not outcome in terms of hospitalisation length, as shown in Fig 3B. We ran model diagnostics for this model, which showed that a linear relationship between age and length of hospital stay is justified.

-	Lines 157ff: See my explanations above about the fact that the patients that get hospitalized at around the critical period (day of illness 4-6) have a higher baseline probability of severe disease.

Thank you for raising this point; we have added this to the Discussion of our revised manuscript.

-	Line 174: Because of the natural history of disease, blood biomarkers should really be presented by ‘day of illness’ (‘or day of illness bins’ if necessary for sample size reasons).

As we have explained in our response to a similar point raised earlier, the purpose here was to show how blood biomarkers at hospital admission differ between severity score 1 and 2 and then how these differences can be explained by taking symptom days into account. We feel that this is an important point to reiterate. Also, figure 4 shows the distribution of some of these biomarkers stratified by symptom days.

-	Line 174ff: It seems the results of the blood biomarkers are presented as univariate. Would it be possible to present a regression with severity score 2 as the outcome?

The problem with presenting the results from a (logistic) regression analysis as suggested is that the various blood markers are highly correlated. The aim here was to show how these markers differ between severity score 1 and 2 *irrespective* of other markers, which is why we chose to present the results from a univariate analysis. Please note also, the emphasis here was not on identifying biomarkers of disease severity (which has been covered extensively in the literature, as cited in our revised manuscript) but length of hospitalisation.

-	Line 199: Could it be that the result that ‘hospitalization length was lower in patients with higher severity score’ is due to the fact that they were ‘further along’ in their natural history of disease, being hospitalized on a later day of illness? The finding itself needs to be interpreted in the context.

Yes, this is exactly our interpretation of our findings, which we also pick up in the Discussion.

-	Line 207: Again, the “traditional non-specific infection markers” should be analyzed adjusting for day of illness.

Please see our response to a similar comment above.

-	Line 228: “early hospitalization” rather than “early diagnosis”?

In the context of this work they are the same, as diagnosis was done when the patients presented to hospital.

-	Line 246ff.: Maybe the different severity mix including the distribution of day of illnesses is partially responsible for the heterogeneity by year shown in figure 5 and the accompanying text?

This is an interesting point. Although the distribution of disease severity was different between 2017, 2018 and 2019, the distribution of illness days before hospital admission was remarkably stable. One difference that we found could potentially contribute to the observed heterogeneity is that the average patient age was significantly lower in 2017 (37 vs 40 and 42 in 2018 and 2019, respectively). We have added this to the revised manuscript.

Figure 3:

-	As mentioned before, it might be interesting to see if there are non-linear trends with regard to age, using splines?

Please see our response to the similar point raised above.

Figure 5:

-	This is an important figure, showing the heterogeneity between years. Would it be good to talk about this heterogeneity in the methods section already, see my comment for table 1?

As mentioned earlier, we have expanded Table 1, which now highlights this inter-annual heterogeneity.

Discussion:

-	Line 276 and line 285: These results are likely to be due to the natural history of disease, which calls for stratifying or adjusting for ‘day of illness’ at time of hospitalization. See my general comments above. This theme is reiterated…

Please see our earlier responses to similar comments.

-	Line 328: The authors mention for the first time that the year 2017 had a large dengue outbreak with on average shorter hospitalization periods. The authors speculate if the hospital capacity was more strained in 2017 and therefore people were discharged earlier than in subsequent years? If yes, this would question their findings substantially as their results are driven by the 75% of patients from 2017. It might be important to do a sub-analysis of 2017 only data to confirm if the trends of the results reported are valid!

Maybe this point in the Discussion was slightly misleading as the average length of hospitalisation only went up from 5.2 days in 2017 to 5.8 days in 2018 and 6.1 days in 2019, so it is highly unlikely that this had any effect on our findings. Equally, as mentioned earlier, the number of symptom days prior to hospitalisation was very similar between the years. Figure 5 clearly demonstrates how much our inferences depend on the year of sampling (Fig. 5 B shows the results of sub-analyses for each individual year), which also shows that one of the most robust indicator for the length of hospitalisation is the duration of symptoms prior to hospital admission. We are therefore confident that the results presented in this manuscript are valid.
